# Pattern design of a liquid metal-based wearable heater for constant heat generation under biaxial strain

**DOI:** 10.1016/j.isci.2023.107008

**Published:** 2023-05-30

**Authors:** Seongmin Jeong, Jinhyeok Oh, Hongchan Kim, Joonbum Bae, Seung Hwan Ko

**Affiliations:** 1 Applied Nano and Thermal Science Lab, Department of Mechanical Engineering, Seoul National University, 1 Gwanak-ro, Gwanak-gu, Seoul 08826, Korea; 2 Bio-Robotics and Control Lab, Department of Mechanical Engineering, Ulsan National Institute of Science and Technology, 50 UNIST-gil, Ulsan 44919, Korea; 3 Institute of Engineering Research/Institute of Advanced Machinery and Design (SNU-IAMD), Seoul National University, Gwanak-ro, Gwanak-gu, Seoul 08826, Korea

**Keywords:** Thermal design, Engineering, Materials science

## Abstract

As the wearable heater is increasingly popular due to its versatile applications, there is a growing need to improve the tensile stability of the wearable heater. However, maintaining the stability and precise control of heating in resistive heaters for wearable electronics remains challenging due to multiaxial dynamic deformation with human motion. Here, we propose a pattern study for a circuit control system without complex structure or deep learning of the liquid metal (LM)-based wearable heater. The LM direct ink writing (DIW) method was used to fabricate the wearable heaters in various designs. Through the study about the pattern, the significance of input power per unit area for steady average temperature with tension was proven, and the directionality of the pattern was shown to be a factor that makes feedback control difficult due to the difference in resistance change according to strain direction. For this issue, a wearable heater with the same minimal resistance change regardless of the tension direction was developed using Peano curves and sinuous pattern structure. Lastly, by attaching to a human body model, the wearable heater with the circuit control system shows stable heating (52.64°C, with a standard deviation of 0.91°C) in actual motion.

## Highlights

• Pattern design for stable heating under biaxial strain
• A simple and rapid fabrication method with liquid metal direct ink writing
• Liquid metal-based wearable heater control system

## Graphical abstract

**Figure:**
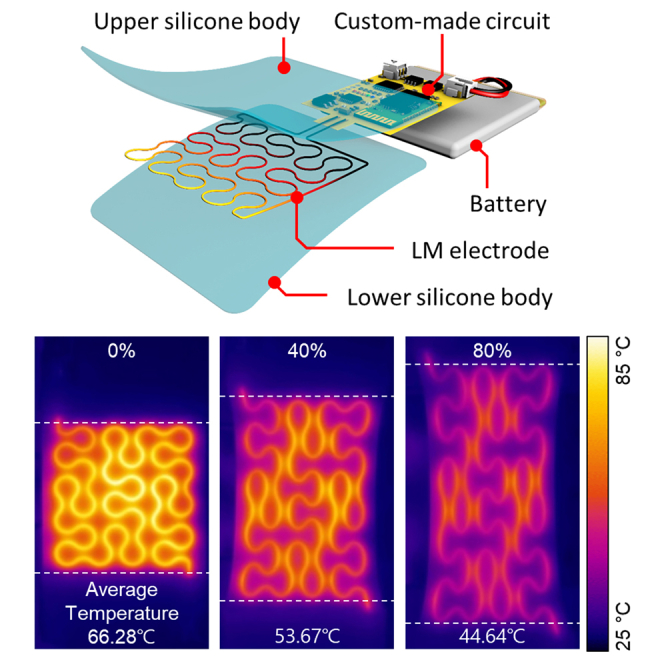

## Introduction

With the advancement of technology, wearable electronics are a technology that is essential for enhancing user convenience and quality of life, and it has been the subject of extensive research and attention for many years.^1^^,^^2^^,^^3^^,^^4^^,^^5^^,^^6^^,^^7^ Researchers are currently exploring ways to enhance the elasticity and tensile stability of wearable devices in various fields, including electrodes, sensors, displays, batteries, and supercapacitors, with the aim of making them more adaptable and robust for practical applications.^8^^,^^9^^,^^10^^,^^11^^,^^12^ Among these, the wearable heater benefits various applications, such as heated clothes, thermal displays, sweat sensors, microchips, and medical devices.^13^^,^^14^^,^^15^^,^^16^^,^^17^^,^^18^ Particularly, the advantages of the transparent heater, such as the ability to monitor the attachment site, improved aesthetics, and visually striking effects, are driving its increased popularity in research.^19^^,^^20^^,^^21^ For this, developing more durable designs, materials, and control systems is crucial.

As the function of a wearable heater is to transfer (heat transfer) the energy produced by the electrode to the target substance or human skin, it is essential to keep the average temperature of the heater constant. Although recent research has explored solar heating and light-thermal reactions using light-reactive or absorbing materials like metal oxides, they cannot be controlled according to the intention of the user, making the Joule-heating method a necessary heat source for these purposes.^22^^,^^23^^,^^24^^,^^25^^,^^26^^,^^27^ In research about resistive heaters for wearable electronics, ensuring the stability and precise control of heating remains challenging due to the physical deformation caused by the human body movement, which can directly affect changes in the area and electrical characteristics in two-dimensional dynamics. Recent research on 2D tensile sensor electrodes with a multi-layer structure, prediction of 2D deformation using deep learning, and heat control has been conducted to improve the problem.^28^^,^^29^^,^^30^ However, there is still a need for the development of heaters with power feedback control systems based on immediate electrical signals to overcome issues such as the complexity of the structure and manufacturing methods, extensive data acquisition and computing process, and accuracy reduction by errors.

Therefore, regardless of the direction of strain, the heater must exhibit the same change when deformed by tension in order to ensure reliability, and there has been a study about patterns and materials of the electrode. Grid patterns and more intricate designs like the Moore curve and Greek cross have been employed. Lately, patterns with Peano curves and fractals have been used as electrode designs for high stability during biaxial tension, but further investigation is needed to apply the presented patterns to the heater, with analysis on the thermal performance according to the patterns.^31^^,^^32^ In materials, composite materials made of indium tin oxide (ITO), carbon nano-materials, and metal nanowires have frequently been used for transparent-stretchable heaters.^33^^,^^34^^,^^35^ But, since these nano-materials are solid, which has ductility and fracture properties, there is a limit to their stability against tension.^36^ Recently, the liquid metal (LM) is becoming popular as an alternative material for stretchable and highly conductive electrodes. LM preserves high electrical and thermal conductivity even when stretched, but it is difficult to produce consistent fluidic channel as designed due to its low viscosity.^37^^,^^38^^,^^39^^,^^40^^,^^41^ Thus, to further advance wearable heater elements, it is necessary to study heating characteristics based on stable patterns in multiaxial strain employing a novel material known as LM.

Here, we report a pattern study for the circuit control of a wearable heater based on LM. The wearable heaters with various patterns were fabricated using the LM direct ink writing (DIW) method, and it was confirmed that the heating characteristics varied depending on the density and orientation of the pattern. It was shown that the input power per unit area determined the average heating temperature. In the case of a directional pattern, the resistance change varied with respect to the tensile direction at 100% strain from 1.12 times to a maximum of 3.76 times the initial value. It implies that the direction of stretching with a directional pattern affects the relationship between the area and the change in resistance. To address this issue, using Peano curves and serpentine pattern structure, the wearable heater that has the same minimum resistance change independent of the tensile direction was developed. In addition, based on the electrical properties of a wearable heater with a Peano-serpentine pattern, the circuit control was used to build a system that can maintain a consistent heating temperature. The wearable heater with the system demonstrates stable heating (52.64°C, with a standard deviation of 0.91°C) in real motion by attaching to a human body model.

The novelty of this work can be summarized as follows: 1) a semi-transparent and highly stretchable soft heater was manufactured by the DIW of LM, 2) design and analysis of the printing patterns of the LM for low resistance change under biaxial strain, 3) a wearable heating device maintaining constant temperature under stretched condition was developed with a simple circuit and control algorithm.

## Results and discussion

### Fabrication process of LM-based wearable heater

**Figure 1:**
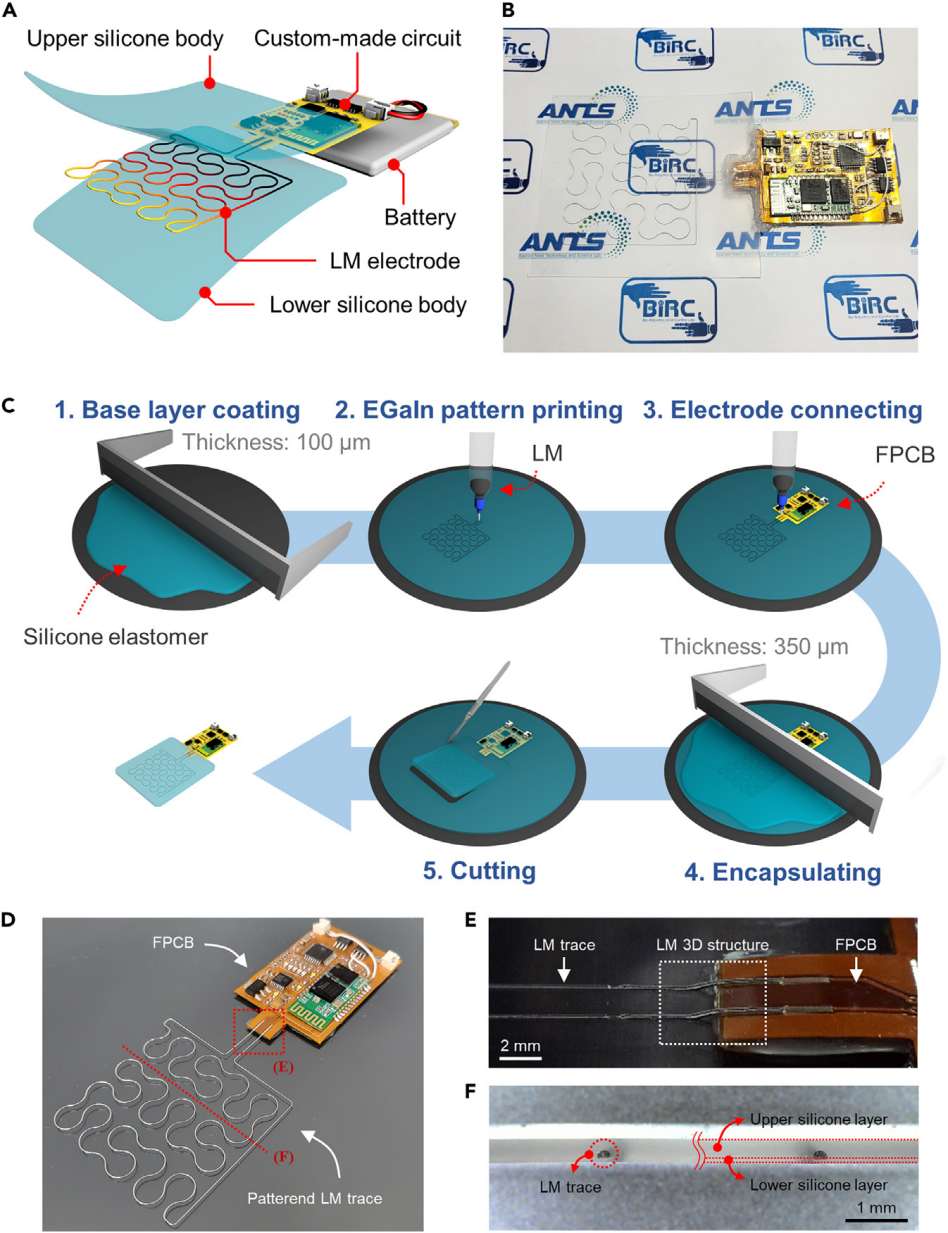
Structure and fabrication process of the wearable heater (A) Components and structure of the wearable heater. (B) Photograpic image of wearable heater with high transparency. (C) Manufacturing process of wearable heater. (D) Photographic image of the patterned LM trace after the electrode connecting step. (E) Microscopic image of the LM 3D structure for electrical connection between the LM trace and FPCB electrodes. (F) Cross-sectional microscope image of the wearable heater.

Figure 1A shows the structure of the proposed wearable heater, which is composed of an LM electrode, a silicone body, and a custom-made circuit for wireless communication. The LM electrode and silicone body generate and transfer heat to the user, respectively.^42^^,^^43^^,^^44^ Eutectic gallium-indium (EGaIn) was selected among various LM materials for its high electrical and thermal conductivity with low toxicity, making it ideal for a high-performance wearable heater.^45^^,^^46^ DragonSkin was used for the silicone body due to its high thermal conductivity, low stiffness, and semi-transparency, as demonstrated in Figure 1B.

The soft wearable heater was fabricated using the process depicted in Figure 1C. First, a layer of silicone with a thickness of 100 μm was coated on a silicon wafer. Next, LM was patterned on the coated silicone using a DIW method. A custom-made flexible printed circuit board (FPCB) was then attached to the silicone layer based on the location of the patterned LM trace. The LM trace and FPCB were connected via a 3D printed structure of LM, as shown in Figures 1D and 1E. To encapsulate the printed LM pattern, a second layer of silicone with a thickness of 350 μm was applied, and any unnecessary silicone was trimmed during the cutting step after the silicone had cured entirely.

To achieve semi-transparency and heating performance, the soft wearable heater was fabricated with a thin thickness of 350 μm. As shown in Figure 1F, the LM trace inside the wearable heater was encapsulated by two silicone layers with thicknesses of 60 and 300 μm, respectively, which were determined through empirical analysis to prevent LM leakage under mechanical stress when the thickness of LM trace was 150 μm. To ensure accurate coating of the silicone layer to each target thickness, a film applicator was utilized. The relationship between the thickness of the silicone layer and the height of the blade in the film applicator is shown in Figure S1 in the supplemental information.

### Investigating the effect of pattern density and direction for heating performance at stretching

**Figure 2:**
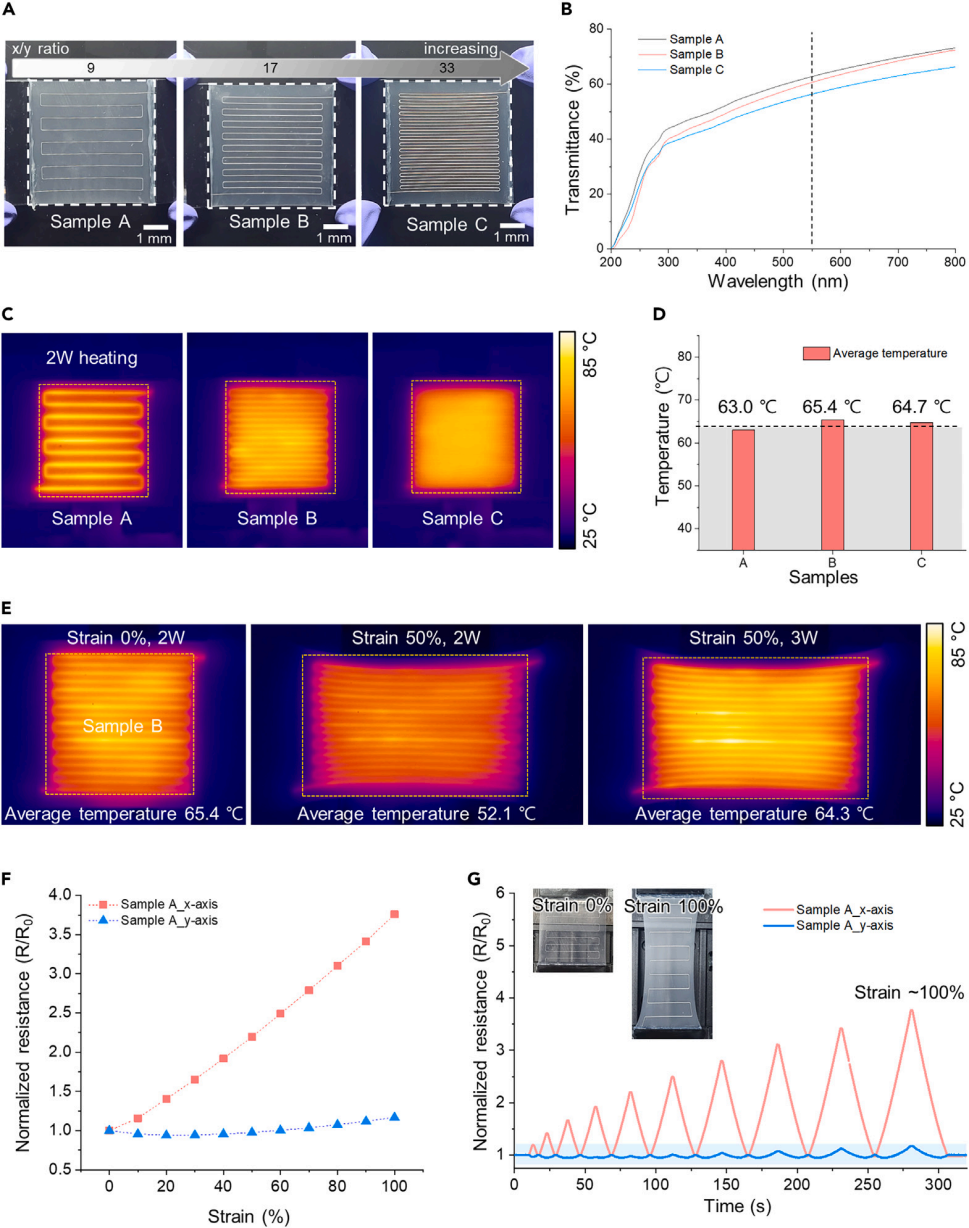
Electric and temperature properties at stretching with density and direction of pattern (A) Photographic image of LM Patterned wearable heater with different x/y ratios. (B) Optical transmittance of the fabricated wearable heater for sample A–C. (C) IR image of heated sample A, B, and C with 2 W input power. (D) Average temperature of heating area when the samples A–C generate heat with 2W input power. (E) IR images of the heated sample B under 0% and 50% strain with different input voltage. (F) Normalized electrical resistance changes of the sample A with stretching under 100% strain. (G) Normalized electrical resistance changes of the sample A with 10 cycles stretching under various strain conditions and returned to its original state.

For the semi-transparent and stretchable wearable heater, the pattern design of the electrode is the most important component to appear with optical, electrical, and electric-heating properties even at stretching conditions. The factors constituting the pattern are diverse; among them, the density and orientation of the pattern have a direct correlation with transparency and stability during strain. For this reason, we examined the effect of pattern design and orientation on the heater performance, even under a stretched condition. Figure 2 shows the electrical characteristics and heating performance of fabricated LM-based wearable heaters with different configuration conditions. The density of LM electrode pattern can be expressed as an x/y ratio, and samples A, B, and C were prepared with different x/y ratios of 9, 17, and 33, respectively, presenting that the higher x/y ratio implies the denser LM electrode. (Figure 2A) The transparency at 550 nm of the samples slightly declines to 62.8, 60.6, and 56.4% (the difference less than 5.4%) as the x/y ratio increases because the LM printing length per unit area (or the cover area) increases. (Figure 2B) Following that, when heating the patterned heater with the same power (∼2 W), the average temperature is similar to 63–65°C. (Figures 2C and 2D) Since the input energy density is equal for the same heating area, the average temperature for every pattern design is approximately ∼64°C. However, at 0% and 50% strain, heating with the same power (2 W) results in average temperatures of 65.4 and 52.1°C, respectively. The reason is that the input power per unit area drops as the heating area increases by 1.5 times. Due to this, raising power to 3 W at 50% strain results in a similar average temperature (64.3°C) with 2 W power at 0% strain. (Figure 2E) As can be seen from this, it is vital to examine how the electrical properties of the heaters alter depending on the direction of tension as the average temperature of the heater is determined by the input energy density per unit area when it heats up concurrently with tension.

The orientation of the pattern as well as the pattern density of the heating electrode has an enormous influence on how electrical characteristics change when stretched in a specific direction. Figure 2F shows the results of the resistance changes with the tensile strain (until ∼100%) in the x and y axis directions of sample A (x/y ratio 9). The x axis directions mean that the line, which consists of a pattern, and the stretching direction are parallel. As shown, the resistance significantly varies as sample A is stretched, changing to 3.76 times the initial values at 100% tensile strain. In this instance, the Poisson’s effect results in the LM electrode in elastomer becoming longer and thinner (Figure 2G inset images), significantly altering resistance as the stretchable heater is stretched. Also, the absolute value of the resistance changes increases with the number of LM pattern lines or density. (Figure S4) In the case of y axis stretching, the resistance maintains within a reliable range (<1.12) even under 100% tensile strain because the pattern and stretching direction are perpendicular. In ten-cycle tests with tension increased by 10% each, Figure 2G shows that steady resistance change can be validated without corrupting the initial values, but the amount of change distinctly differs regarding the x axis and y axis orientations. Thus, as the directionality of the pattern causes the change in resistance to vary substantially depending on the tensile direction, it is essential to improve the design to reduce it.

### Electric properties and electric-heating performance with Peano curves and serpentine design

**Figure 3:**
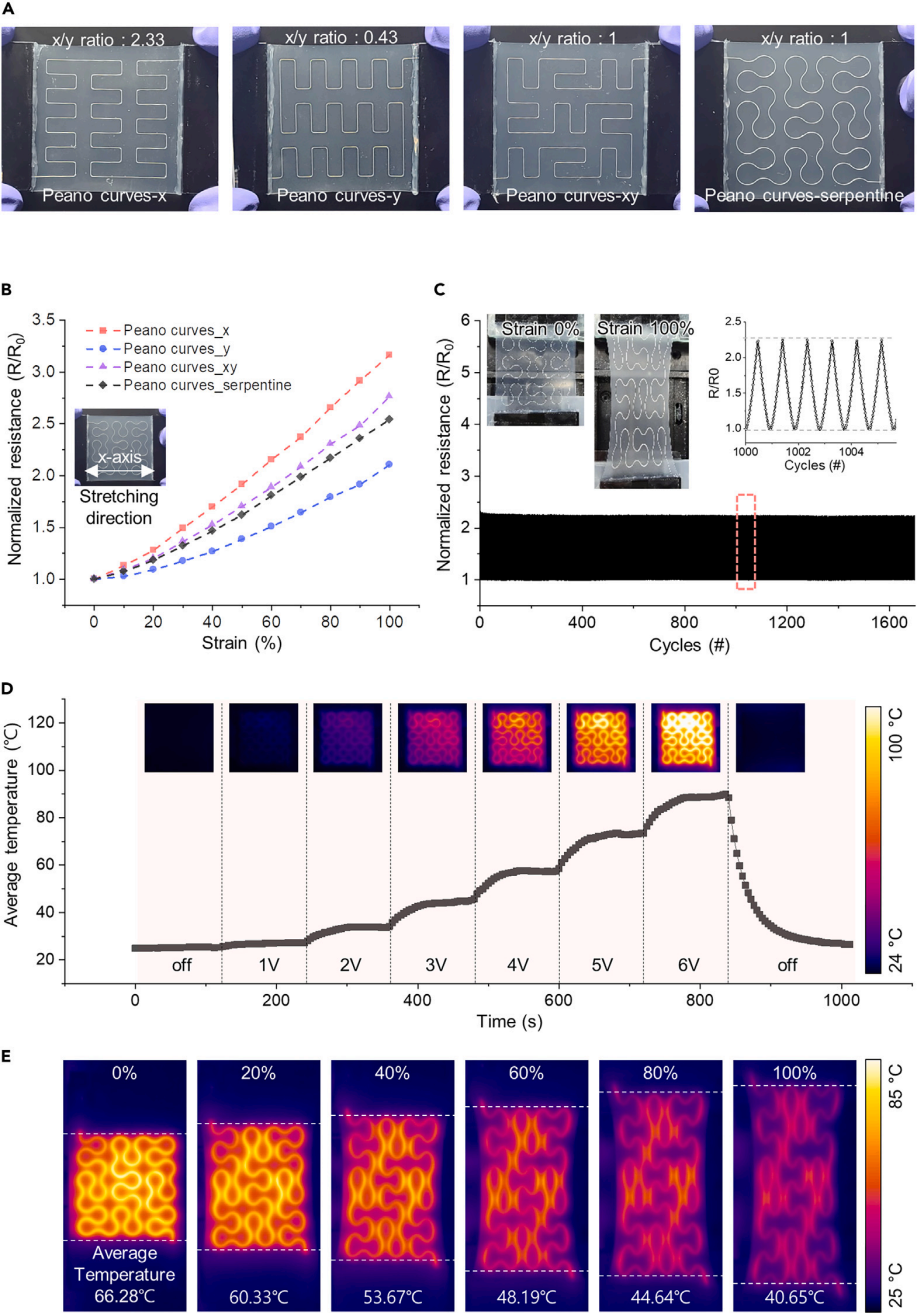
The Peano-serpentine patterned LM wearable heater (A) Photographic image of the wearable heaters with various Peano curves and serpentine pattern. (B) Normalized electrical resistance changes of the Peano curves and serpentine patterned heaters with stretching under 100% strain. (C) Mechanical stability test for the Peano-serpentine patterned heater measured by repeated 100% strain over 1700 cycles. (D) IR images and average temperature change as a result of the increasing voltage up to 6V without stretching. (E) IR images and average temperature under increasing strain during heating.

In order to run a wearable heater steadily, a robust design in a dynamic stress environment is required since most human joints have two or more degrees of freedom. This study applied Peano curves and serpentine structures to the LM-based highly stretchable and semi-transparent wearable heater to study electric characteristics and thermal stability according to the x/y ratio and tensile direction. Depending on the directionality, Peano curves patterns have various designs with different x/y ratios. Figure 3A depicts the samples fabricated for this investigation, which correspond to x/y ratios of 2.33 (Peano curves-x), 0.43 (Peano curves-y), and 1 (Peano curves-xy), respectively. The electrical and thermal properties can be compared and analyzed according to the stretching direction of the Peano curves design pattern with directionality (x/y ratio is not 1) through Peano curves-x and Peano curves-y samples. As shown in Figure 3B, the change in resistance at 100% stretching in Peano curves-x and Peano curves-y corresponds to 3.17 and 2.11 times the initial value, respectively, and more of a change is seen in Peano curves-x than Peano curves-y. It implies that the increase in electrical characteristics during stretching in the x axis direction is proportional to the x/y ratio, and resistance stability decreases with strain in a certain direction. In comparison, the Peano curves-xy with an x/y ratio of 1 exhibit 2.76 times at 100% strain, which is the median value of these two, but shows the same change whether stretched in the x axis or y axis directions. (Figure S5) As a result, the Peano curves-xy design is more stable for the wearable heater with circuit control in various in-plane tensile situations. Furthermore, the serpentine design was applied to reduce changes in resistance during tension. Consequently, the reduction of 7.97% to 2.54 times the initial value at equal strain can be verified. (Figures 3A and 3B).

As was already discussed, the area and input energy determine the heating properties of a heater. The area and resistance alter when the wearable heater is stretched, and these two changes must be recognized to govern temperature stabilization through circuit control. For this, it is necessary to know the change in the area only by changing the electrical characteristics. But, if the resistance varies differently depending on the direction with the same strain, it is unknown how much power will be required to achieve the desired temperature. Therefore, a heater design that exhibits the same minimum resistance change for strain in both the x and y axis directions is necessary. Through the design, heat generation can be controlled by altering electrical properties alone, regardless of the strain direction. For this reason, Peano curves-serpentine is suitable as a pattern design for wearable heaters with circuit control.

To confirm the mechanical stability of the Peano curves-serpentine design samples, as shown in Figure 3C, 100% tensile tests were repeated for more than 1700 cycles. Throughout the experiment, there was no decline in conductivity, and as appears in the inset of Figure 3C, stable changes persisted after 1000 cycles. The degree of resistance change at 100% strain remains at 2.23 even after 1700 cycles. Also, Figure S6A demonstrates that the electrical characteristics remain consistent when the tension is kept at a specific strain.

In addition, the temperature was verified by providing power without control using a circuit to evaluate the heating performance of the patterned electrode. In Figure 3D, as the voltage was raised gradually by 1 V up to 6 V, the temperature varied, as shown in the inset infrared (IR) images. The average temperature increases up to ∼89°C (at 6 V), and the change of electric properties is negligible when examining the resistance in relation to temperature, as illustrated in Figure S6B. Also, a constant voltage was applied with strain (∼100% strain) to evaluate the average temperature of the heater during tension. (Figure 3E) Due to the increase in area and decrease in resistance caused by strain, the average temperature drops from 66.28 to 40.65°C. It is feasible to supplement heat generation by circuit control in this design as, regardless of the direction of strain, the correlation between area and resistance on stretching is determined.

### Circuit design for power control applied to heater and performance verification

**Figure 4:**
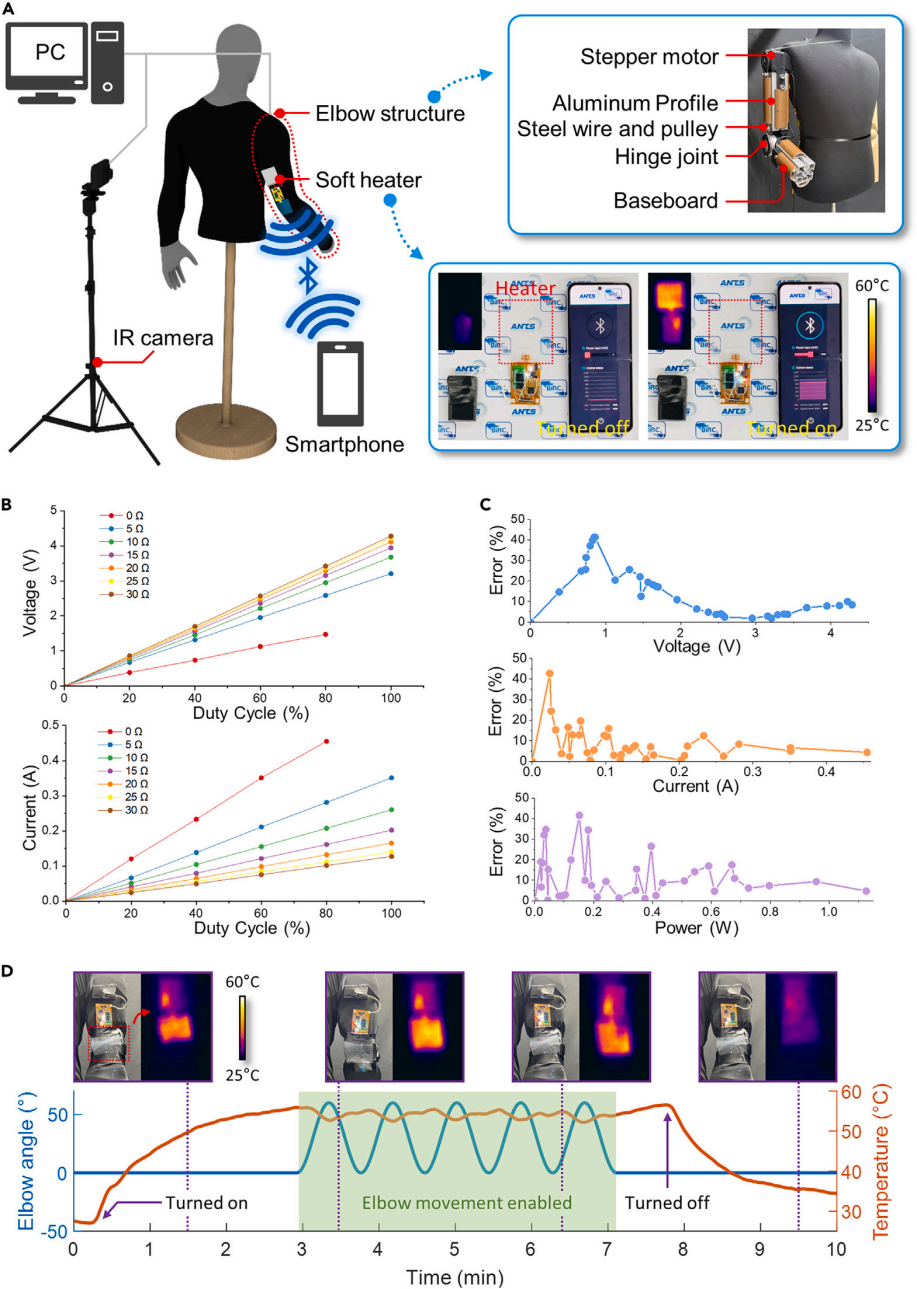
Circuit control design and human body model application (A) Graphic image of the experimental setup with a smartphone app for power control of the heater, an IR camera for temperature measurement, and a mannequin structure that can reproduce human elbow movement. (B) Changes in applied voltage and current to the heater observed in response to variations in duty cycle and heater resistance. (C) The measurement accuracy varied with changes in circuit voltage, current, and power. (D) Heater temperature and joint angle in response to movement of the mannequin’s elbow joint while 1 W of power was applied to the heater, along with photographic and IR camera images of the heater.

To verify the performance of the proposed soft heater as a wearable device, a test setup in Figure 4A was used. The effect of changes in human motion on wearable heaters was analyzed by mimicking the elbow joint movement using an elbow structure of a mannequin. The stepper motor on the shoulder transmitted torque to the hinge joint located at the elbow joint via steel wire and pulleys. Aluminum profiles and baseboards were used to represent the bones and muscles of the human body. The soft heater attached to the clothing on the mannequin was controlled to maintain the power set by a smartphone, while an IR camera measured its surface temperature. The elbow structure and IR camera were connected to a personal computer (PC) through wire communication for movement control and data measurement. The wearable heater was wirelessly connected to a smartphone to transmit and receive data on the target and apply power to the heater. The inset in Figure 4A demonstrates the temperature control of the heater via a smartphone.

To measure the applied power to the heater, a circuit capable of real-time voltage and current measurement for constant power was designed. To evaluate the performance of the circuit, voltage and current changes were measured as the heater resistance increased from 5 Ω to 35 Ω when the duty cycle of the pulse-width modulation (PWM) signal to the motor driver was adjusted in increments of 20% from 0% to 100%, except for 5 Ω due to output limit. As the resistance of the heater decreases, a decline in voltage, even at a duty cycle of 100%, was observed from 4.279 V to 3.207 V, as shown in the first graph of Figure 4B. On the other hand, as the resistance decreases, the current increases from 0.140 A to 0.455 A, as shown in the second graph of Figure 4B. However, due to the greater voltage drop, the power applied was 1257 mW at the lowest resistance of 5 Ω, while it was only 543 mW at the highest resistance of 35 Ω. Figure 4C shows the accuracy of voltage, current, and power measurements. The percent error increased with decreasing power due to noise, with the maximum and average percentage errors in power measurement being 12.73% and 6.83%, respectively, for a power input range of 500 mW or higher, capable of heating the heater over 45°C.

To maintain consistent heating performance under human movements, a power control system was applied to the wearable heater. By measuring the voltage and current in the FPCB circuit and transmitting the data to a smartphone app, the app calculated the control output based on the applied power using a proportional-integration-differential (PID) control algorithm, which was then sent back to the circuit to apply the target power to the heater (Video S1). A heater was attached to the elbow joint of a mannequin to verify the heating performance of the proposed control method. The temperature variation was measured by IR camera as the joint angle was repetitively changed from 0° to 60° while maintaining a target power of 1W (Video S2). As shown in Figure 4D, during the first 3 min, the elbow joint was kept at a constant angle, and the graph shows that the maximum temperature of the heater was 55.84°C within 165 s of being turned on. For the next 4 min, the angle of the elbow joint varied from 0° to 60°, and the average temperature of the heater was 54.18°C with a standard deviation of 0.94°C, which indicated that the suggested control method was successful in maintaining the target temperature with a small amount of error.

Video S1. Controlling power applied to the heater by a smartphone app

Video S2. Control of a wearable heater control under elbow movement

### Conclusion

This paper proposes a wearable heater that can be utilized in daily life and medical purposes. The heater was made of silicone and LM, which have high thermal conductivity and stretchability. LM was patterned using the DIW method to produce traces for heat generation and connecting wires for electrical connection. To ensure stable heating performance under stretched condition, the resistance and temperature changes of several patterns under tension were measured. As a result, Peano curves-serpentine pattern showed the least resistance and temperature variation when stretched in various directions. To verify the temperature maintenance performance of the wearable heater, a test environment that can reproduce human elbow movement using a mannequin with a built-in motor was proposed with a circuit that can maintain the target power of the heater through real-time voltage and current measurement. The experimental results showed that the wearable device could maintain a constant temperature even with a 40° change in elbow angle, suggesting its potential use as a compact wearable device for heat therapy, rehabilitation, and daily temperature maintenance.

### Limitations of the study

This research shows the design and control system for reliable temperature in a 2D tension with the LM-based wearable heater. However, since various deformations, such as twists and pressure, occur together in the actual wearable device environment, additional research related to this is needed. In addition, heat transfer and control issues arise due to variations in physical properties between the heating substance and the human body. Therefore, we plan the subsequent research for conformal contact by improving the device.

## STAR★Methods

### Key resources table

**Table:** 

REAGENT or RESOURCE	SOURCE	IDENTIFIER
**Chemicals, peptides, and recombinant proteins**		

Gallium-Indium eutectic (EGaIn)	Sigma-Aldrich	CAS: 7440-55-3 (Gallium) CAS: 7440-74-6 (Indium)
DragonSkin 30	Smooth-on	https://www.smooth-on.com/products/dragon-skin-30/
Silicone rubber (Sil-poxy)	Smooth-on	https://www.smooth-on.com/products/sil-poxy/

**Software and algorithms**		

Unity (version 2020.3.20f1)	Unity Technologies	https://unity.com/

### Resource availability

#### Lead contact

Further information and requests for resources and reagents should be directed to and will be fulfilled by the lead contact, Prof. Seung Hwan Ko (maxko@snu.ac.kr).

#### Materials availability

This study did not generate new unique reagents.

### Method details

#### Materials

A liquid metal for heat generation was used with EGaIn (Sigma-Aldrich., USA). Silicone elastomer, Dragonskin 30 (Smooth-On, USA), was used to encapsulate the EGaIn. The Dragonskin 30 was mixed in a 1:1 ratio of base to curing agent. To prevent delamination between the FPCB and the silicone body, a reinforcing adhesive using silicone glue, Sil-Poxy (Smooth-On, USA), was applied. The FPCB substrate was ordered and manufactured through PCBWay, China.

#### Fabrication of LM-based wearable heater

First, a silicone release agent (Mann Release Technologies, USA) was sprayed onto a square glass wafer to ensure the easy detachment of the silicone elastomer. Next, the silicone elastomer was coated onto a square glass wafer using a film applicator (Mitutoyo) with a height of 2000 μm and cured at 80°C in an oven for 10 minutes, where the silicone elastomer was prepared by mixing the base and curing agent in a 1:1 ratio without any additives. After the curing process, the FPCB was attached onto the cured silicone layer using silicone adhesive, Sil-Poxy and cured for 5 minutes at 80°C in an oven. For direct ink writing of liquid metal, a 3cc barrel with a tip of internal diameter as 0.40mm (TPND-22G, Musashi) loaded with EGaIn was mounted on a three-axis motion platform (Omega X, Musashi). The printing tip was controlled to move at a speed of 3 mm/s during EGaIn trace printing and 0.3 mm/s during 3D structure printing for FPCB connection, with a 70 μm stand-off distance from the coated silicone layer. Instead of applying pressure during printing, EGaIn was extruded by its weight due to the formation of an oxide layer. At the end of printing process, the tip was moved at a speed of 50 mm/s to stop extrusion by shear force. When the direct ink writing of liquid metal was completed, the encapsulating layer was coated using the film applicator with a height of 2450 μm and cured at 80°C in an oven for 10 minutes. The silicone elastomer was cut to a size of 60 mm in width and 70 mm in length using a surgical knife after curing process. Since the bonding area between the silicone elastomer and the FPCB is vulnerable, Sil-poxy was applied to the boundary between silicone and FPCB after cutting process.

#### Circuit design and experiment setup

A custom-made flexible printed circuit board (FPCB) was designed with a small form factor (55mm x 33mm) for a conformable wearable heating device. The FPCB consisted of a ATMEGA 328P as (ATMEL, USA) microcontroller unit (MCU), a HC-06 Bluetooth module (Shenzhen HC Technology, China) HR8833 motor controller, ACS712 current meters (Allegro MicroSystems, USA), and LM358 operational amplifiers (Texas Instruments, USA). To measure the power applied to the soft heater, a differential amplifying circuit and a current meter circuit were used to measure voltage and current, respectively. As both the input and output of the motor driver were PWM signals, a resistor-capacitor low-pass filter was employed to measure the current and voltage via the MCU.

## Data Availability

All data reported in this paper will be shared by the lead contact upon request. This paper does not report original code. Any additional information required to reanalyze the data reported in this paper is available from the lead contact upon request.
