# Supplementary material for: Pattern design of a liquid metal-based wearable heater for constant heat generation under biaxial strain
Source: iScience. 2023 May 30;26(7):107008. doi: 10.1016/j.isci.2023.107008 (PMC10275728; doi:10.1016/j.isci.2023.107008)
Supplement: Document S1. Figures S1–S8 [file mmc1.pdf]

iScience, Volume *26*

Supplemental information

Pattern design of a liquid metal-based wearable heater for constant heat generation under biaxial strain

Seongmin Jeong, Jinhyeok Oh, Hongchan Kim, Joonbum Bae, and Seung Hwan Ko

Pattern Design of a Liquid Metal Based Wearable Heater for Constant Heat Generation under Biaxial Strain

Seongmin Jeong[1,4], Jinhyeok Oh[2,4], Hongchan Kim[1], Joonbum Bae[2], Seung Hwan Ko[1,3,5]**

[1] Applied Nano and Thermal Science Lab, Department of Mechanical Engineering, Seoul National University, 1 Gwanak-ro, Gwanak-gu, Seoul 08826, Korea
[2] Bio-Robotics and Control Lab, Department of Mechanical Engineering, Ulsan National Institute of Science and Technology, 50 UNIST-gil, Ulsan 44919, Korea
[3] Institute of Engineering Research / Institute of Advanced Machinery and Design (SNU-IAMD), Seoul National University, Gwanak-ro, Gwanak-gu, Seoul, 08826, Korea
[4] These authors contributed equally
[5] Lead contact
[*] Correspondence : jbbae@unist.ac.kr, maxko@snu.ac.kr

[*] To whom correspondence should be addressed.
E-mail: jbbae@unist.ac.kr, maxko@snu.ac.kr

* None of the material has been published or is under consideration elsewhere, including the Internet.

The number of display items: 4 main figures and 7 supplementary figures

The number of attachments: 1 manuscript, 1 supplementary information, 2 movie clips

Supplemental Information

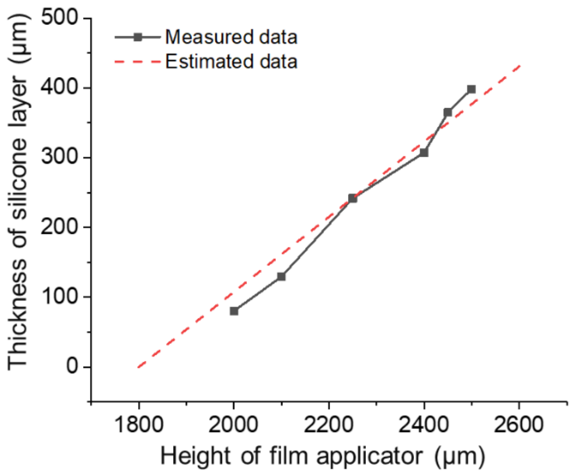

Figure S1. Geometric properties of the fabricated LM-based wearable heater, related to Figure 1.

The relationship between the thickness of the silicone layer and the height of the blade in the film applicator. Since the thickness of the glass wafer used in the coating process was 1800 μm, the thickness of the silicone layer was 0 μm when the height of the film applicator was set to 1800 μm. The R^2 value of the regression model is 0.97.

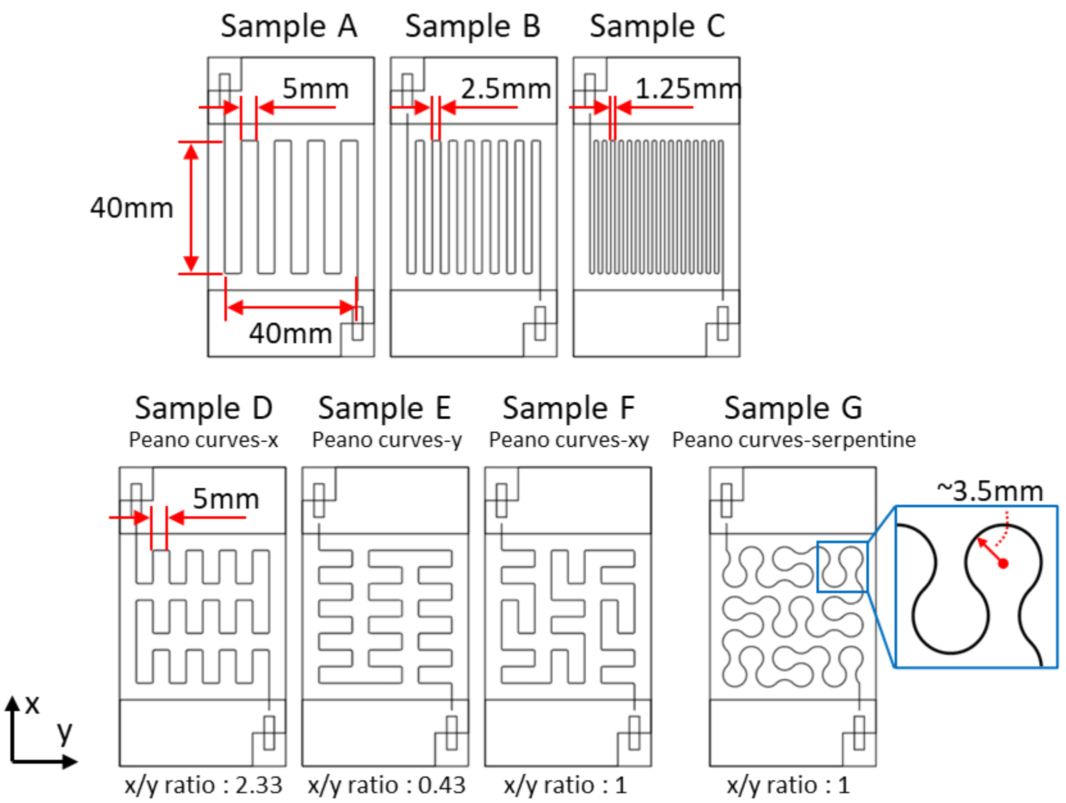

Figure S2. Characterizations of printed LM features, related to Figure 2, 3.

For the same heating area, all samples were produced in a size of 40mm x 40mm. Sample A and B and were printed using a continuous line pattern with a gap of 5mm, 2.5mm, and 1.25mm, respectively, to analyze the changes in resistance, heat distribution, and average temperature as the wire density varies. Samples D, E, and F were printed with the same 5mm gap but with different x/y ratios. Samples F and G were printed with the same 5mm gap and an x/y ratio of 1, but G was printed with a serpentine line with a radius of about 3.5mm.

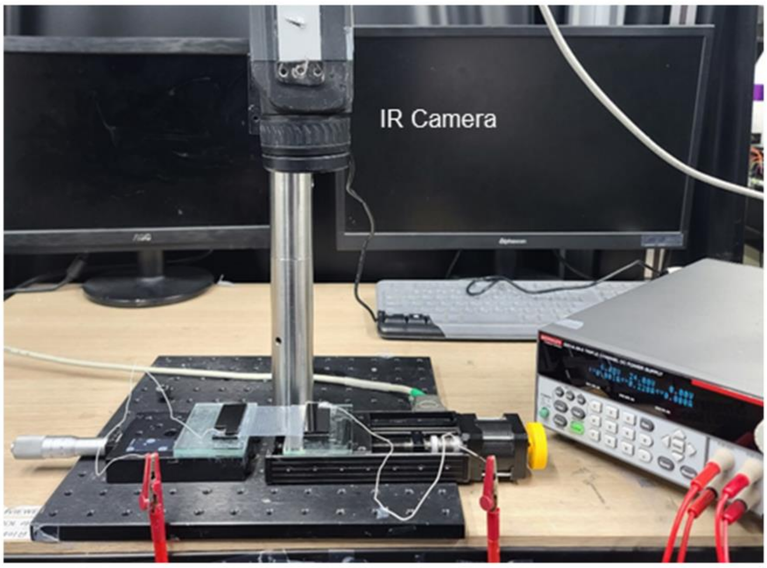

Figure S3. Measurement setup for mechanical, electrical, and heating properties of the heaters, related to Figure 2, 3.

The developed heaters are connected to the power supply and digital multimeter for heating and electrical properties. The heaters were attached to a stretching system with a precise linear stage for the mechanical test. The IR camera was set up to focus on the surface of the heaters.

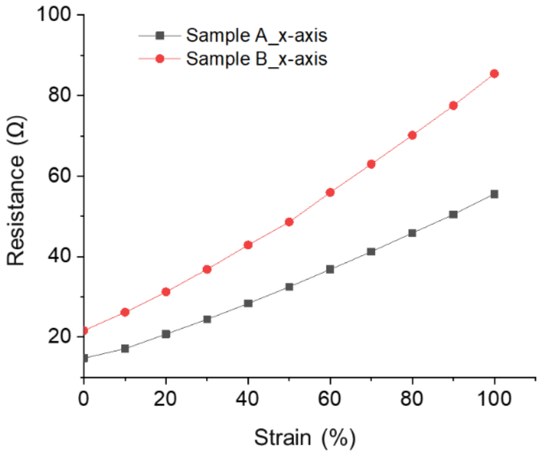

Figure S4. Resistance of samples A and B with x-axis strain, related to Figure 2.

Without normalization of resistance, there was a significant difference in the resistance changes between samples A and B on x-axis stretching.

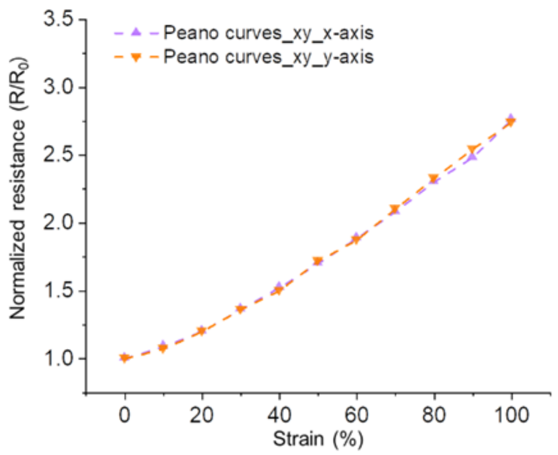

Figure S5. Normalized resistance of the peano curves patterned heater with x/y ratio one on the x-axis and y-axis strain, related to Figure 3.

The peano curves patterned heater with x/y ratio 1 shows the same electric properties change regardless of stretching direction (x-axis or y-axis).

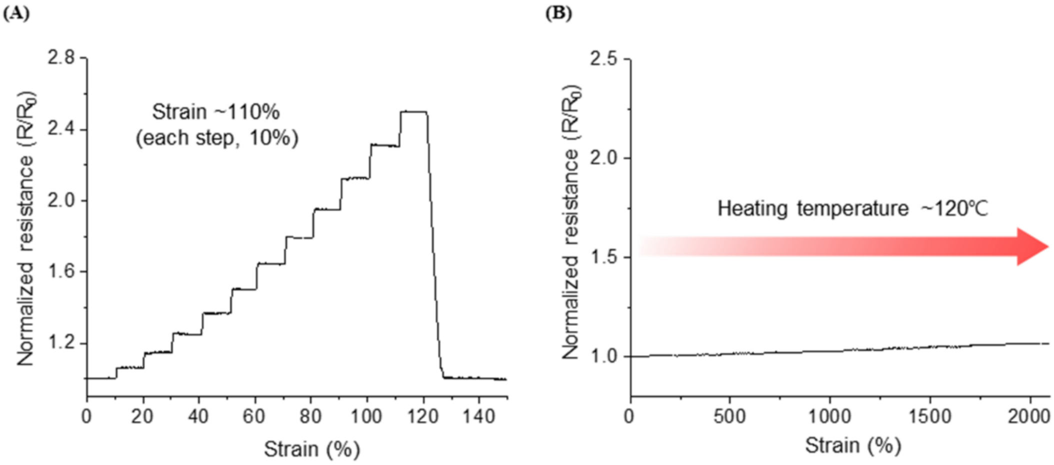

Figure S6. Mechanical and thermal stability of the peano curves-serpentine patterned heater, related to Figure 3.

(A) The peano curves-serpentine patterned heater was stretched up to 110% in increments of 10% stepwise. (B) The temperature of the patterned heater was increased to 120 °C, and it was confirmed that the change in normalized resistance was insignificant.

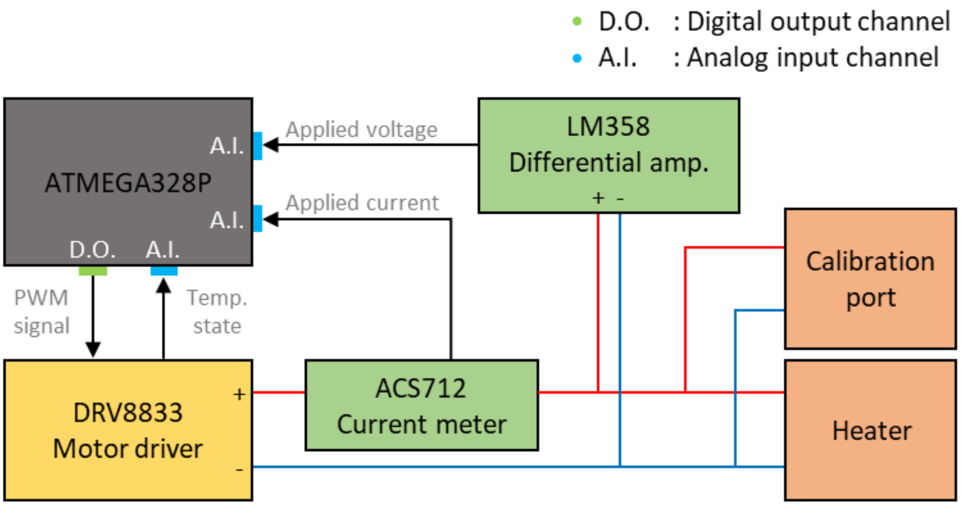

Figure S7. Circuit configuration for power control of the heater, related to Figure 4.

The motor driver was controlled with a PWM signal transmitted from the ATMEGA328P microcontroller, while the over-temperature and over-current states of the motor driver were measured via an analog input channel. The current and voltage applied to the heater were measured through an ACS712 current meter and differential amplifier composed of a LM358 operational amplifier. To establish the functional relationship between the actual voltage and current values and the corresponding analog input signals, a calibration process was conducted by connecting resistors with different resistance values to the calibration port. Once calibration was completed, the resistors were removed, and the heater was connected to the circuit through LM printing process.

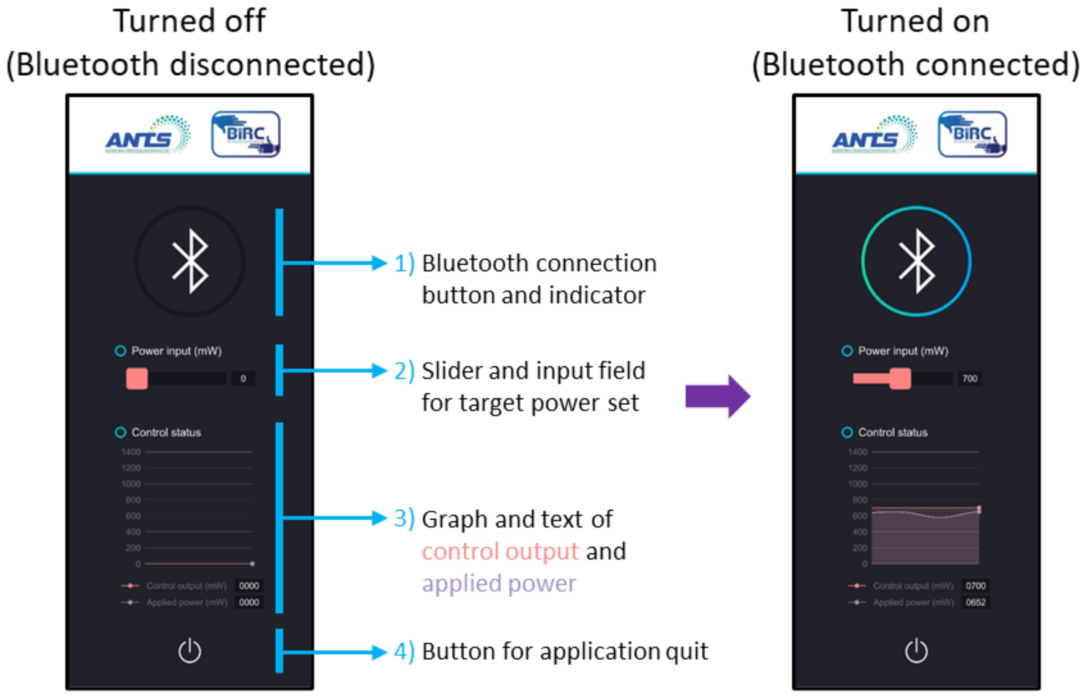

Figure S8. A Unity-based smartphone application for Bluetooth connection and heater control, related to Figure 4.

The application is divided into four parts: 1) a section for Bluetooth connection and connection status indication, 2) a section for setting the target power of the heater, 3) a section for monitoring the power applied to the heater and the control output, and 4) a section for exiting the application. When a socket is successfully created and connected using the name of the Bluetooth module, the circle around the Bluetooth logo is filled with green and blue color, indicating a successful connection. The target power set by the slider and input field is transmitted in an 11-byte packet, which includes delimiters and LED level indications, at the fastest loop speed with an interval of approximately 5 ms. The state of the motor driver, applied voltage, and current are also received at the same speed and in a 16-byte packet including delimiters.
